# An *N*‐Ethyl‐*N*‐Nitrosourea (ENU) Mutagenized Mouse Model for Autosomal Dominant Nonsyndromic Kyphoscoliosis Due to Vertebral Fusion

**DOI:** 10.1002/jbm4.10033

**Published:** 2018-03-08

**Authors:** Christopher T Esapa, Sian E Piret, M Andrew Nesbit, Gethin P Thomas, Leslie A Coulton, Orla M Gallagher, Michelle M Simon, Saumya Kumar, Ann‐Marie Mallon, Ilaria Bellantuono, Matthew A Brown, Peter I Croucher, Paul K Potter, Steve DM Brown, Roger D Cox, Rajesh V Thakker

**Affiliations:** ^1^ Academic Endocrine Unit Radcliffe Department of Medicine University of Oxford Oxford Centre for Diabetes, Endocrinology and Metabolism Churchill Hospital Headington UK; ^2^ MRC Mammalian Genetics Unit and Mary Lyon Centre MRC Harwell Institute Harwell Science and Innovation Campus Harwell UK; ^3^ School of Biomedical Sciences Ulster University Coleraine UK; ^4^ Institute of Health and Biomedical Innovation Queensland University of Technology Translational Research Institute Princess Alexandra Hospital Brisbane Australia; ^5^ Charles Sturt University Boorooma Street Wagga Wagga Australia; ^6^ The Mellanby Centre for Bone Research University of Sheffield Sheffield UK; ^7^ Instituto de Medicina Molecular (IMM) Faculdade de Medicina de Universidade de Lisboa Lisboa Portugal; ^8^ Garvan Institute for Medical Research Sydney Australia

**Keywords:** GENETIC ANIMAL MODELS, DISEASES AND DISORDERS OF/RELATED TO BONE, DXA, PRECLINICAL STUDIES, BONE QCT/µCT

## Abstract

Kyphosis and scoliosis are common spinal disorders that occur as part of complex syndromes or as nonsyndromic, idiopathic diseases. Familial and twin studies implicate genetic involvement, although the causative genes for idiopathic kyphoscoliosis remain to be identified. To facilitate these studies, we investigated progeny of mice treated with the chemical mutagen *N*‐ethyl‐*N*‐nitrosourea (ENU) and assessed them for morphological and radiographic abnormalities. This identified a mouse with kyphoscoliosis due to fused lumbar vertebrae, which was inherited as an autosomal dominant trait; the phenotype was designated as hereditary vertebral fusion (HVF) and the locus as *Hvf*. Micro–computed tomography (μCT) analysis confirmed the occurrence of nonsyndromic kyphoscoliosis due to fusion of lumbar vertebrae in HVF mice, consistent with a pattern of blocked vertebrae due to failure of segmentation. μCT scans also showed the lumbar vertebral column of HVF mice to have generalized disc narrowing, displacement with compression of the neural spine, and distorted transverse processes. Histology of lumbar vertebrae revealed HVF mice to have irregularly shaped vertebral bodies and displacement of intervertebral discs and ossification centers. Genetic mapping using a panel of single nucleotide polymorphic (SNP) loci arranged in chromosome sets and DNA samples from 23 HVF (eight males and 15 females) mice, localized *Hvf* to chromosome 4A3 and within a 5‐megabase (Mb) region containing nine protein coding genes, two processed transcripts, three microRNAs, five small nuclear RNAs, three large intergenic noncoding RNAs, and 24 pseudogenes. However, genome sequence analysis in this interval did not identify any abnormalities in the coding exons, or exon‐intron boundaries of any of these genes. Thus, our studies have established a mouse model for a monogenic form of nonsyndromic kyphoscoliosis due to fusion of lumbar vertebrae, and further identification of the underlying genetic defect will help elucidate the molecular mechanisms involved in kyphoscoliosis. © 2018 The Authors. *JBMR Plus* is published by Wiley Periodicals, Inc. on behalf of the American Society for Bone and Mineral Research

## Introduction

Kyphosis is a common disorder of the vertebral column^1^ that can occur in isolation, or in association with scoliosis in infants^2^ and adolescents,^3^ and osteoporosis in the elderly,^1^ causing pain, decreased function and activity,^1^ and increased risk of mortality in older women above the age of 65 years.^1^, ^4^ Kyphosis, scoliosis, or kyphoscoliosis, can occur at any age, secondary to other underlying developmental, musculoskeletal, neuromuscular, or spinal disorders,^5^, ^6^, ^7^, ^8^, ^9^, ^10^ and may be part of complex disorders such as the CHARGE syndrome (coloboma of the eye, heart defects, atresia of the nasal choanae, retardation of growth and/or development, genital and/or urinary abnormalities, and ear abnormalities and deafness),^9^, ^11^ or occur as a nonsyndromic condition. Indeed, the most common forms of kyphosis and scoliosis in adolescents are nonsyndromic and include: Scheuermann disease^3^ a form of nonsyndromic kyphosis, which affects >8% of the population^12^; idiopathic scoliosis (IS), which affects approximately 2% to 3% individuals^13^, ^14^, ^15^, ^16^; and congenital nonsyndromic scoliosis, which is reported to have a prevalence of approximately 0.5 to 1 per 1000 individuals.^9^ Familial and twin studies have indicated a genetic basis for kyphosis^17^, ^18^, ^19^, ^20^ and scoliosis,^21^, ^22^, ^23^, ^24^, ^25^, ^26^, ^27^, ^28^ with likely genetic heterogeneity. However, studies aimed at defining the genetic abnormalities causing these spinal disorders have been hampered by their phenotypic and genetic heterogeneity, variable modes of inheritance,^8^, ^29^ and gene‐environment interactions that may modify the phenotypic expression.^9^ To facilitate these studies, we investigated the progeny of mice treated with the chemical mutagen *N*‐ethyl‐*N*‐nitrosourea (ENU),^30^ which is an alkylating agent that induces mutations in DNA at a frequency of 1 in every 1.5 megabases (Mb).^31^ These mutations consist mainly of SNPs, and occasionally small indels, but not large structural variants.^31^ Similar approaches using phenotypic assessments of mice with ENU mutations has successfully identified mouse models for hereditary human disorders, including skeletal dysplasias.^32^, ^33^, ^34^ Here, we report an ENU mouse mutant with kyphoscoliosis due to lumbar vertebral fusion.

## Materials and Methods

### Ethics statement

All animal studies were carried out using guidelines issued by the Medical Research Council (MRC) (UK) in “Responsibility in the Use of Animals for Medical Research” (July 1993) and Home Office Project License Number 30/2433. Experiments were also approved by the MRC Harwell ethics committee.

### Generation of mutant mice

Male C57BL/6J mice were treated with ENU and mated with untreated C3H/HeH female mice,^31^ and the resulting progeny were screened at 12 weeks of age for autosomal dominant phenotypes.^34^ In vitro fertilization was used to generate progeny, using methods previously described.^35^ Mice were fed an expanded rat and mouse no. 3 breeding diet (Special Diets Services, Witham, UK) containing 1.15% calcium, 0.82% phosphate, and 4088.65 units/kg vitamin D, and given water *ad libitum*.

### Phenotype assessment, radiography, and DXA

Mice were assessed for dysmorphology as described (European Mouse Phenotyping Resource of Standardised Screens [EMPReSS]).^36^ Anaesthetized mice were assessed by digital radiography at 26 kV for 3 s using a Faxitron MX‐20 digital X‐ray system (Faxitron X‐ray Corporation, Lincolnshire, IL, USA)^34^ and DXA using a Lunar PIXImus densitometer (GE Healthcare, Chalfont St Giles, UK), as reported.^37^ X‐ray images were processed using the DicomWorks software (http://www.dicomworks.com/) and DXA images were processed using the PIXImus software.^37^

### Micro–computed tomography analysis

Formalin‐fixed skeletons and dissected bones were examined using a micro–computed tomography (μCT) scanner (model 1172a; Skyscan/Bruker, Kontich, Belgium) at 50 kV and 200 µA utilizing a 0.5‐mm aluminum filter and a detection pixel size of 4.3 µm^2^ (tibias and lumbar vertebrae) and 17.4 µm^2^ (spinal columns and rib cages). For each specimen, images were captured every 0.7 degrees through a 360‐degree rotation. The lumbar vertebrae were scanned separately to measure trabecular bone,^38^ using a detection pixel size of 4.3 µm^2^, and images were scanned every 0.7 degrees through a 180‐degree rotation.^34^ Scanned images were reconstructed using Skyscan NRecon software and analyzed using the Skyscan CT analysis software (CT Analyser v1.8.1.4; Skycan).^34^ Total bone volume (mm^3^) and bone mineral density (g/cm^3^) were measured over the entire volume of the bone (CT Analyser v1.8.1.4; Skycan). Trabecular bone volume as proportion of tissue volume (BV/TV, %), trabecular thickness (Tb.Th, mm × 10^−2^), trabecular number (Tb. N, mm^−1^), and structure model index (SMI) were assessed for the first and second lumbar vertebrae, using the CT analysis software.^34^ Intact vertebral columns were modeled using Skyscan CT volume software and images captured (CT Vol: Realistic 3D‐Visualization v1.11.0.2; Skyscan). Cross‐sections of lumbar vertebrae and tibias were generated using Skyscan CT analysis software.

### Histology

Dissected vertebrae were fixed in 10% formalin, decalcified in formical‐4 (Decal Chemical Corporation, Suffern, NY, USA) for 3 days before embedding in paraffin wax.^34^ Sections (3 to 4 μm) were cut using a Leica Microsystems Microtome (Leica Microsystems, Milton Keynes, UK) and stained with hematoxylin and eosin (H&E). Slides were examined using a Leica microscope model DM4000B (Leica Microsystems) and images captured using a QImaging camera model 10‐RET‐OEM‐F‐CLR‐12 (QImaging, Surrey, BC, Canada).^34^

### Plasma biochemistry

Blood samples were collected from the lateral tail vein of mice that had fasted for 4 hours. Plasma samples were analyzed, using a Beckman Coulter AU680 semi‐automated clinical chemistry analyzer (Beckman Coulter, High Wycombe, UK), for total calcium, phosphate, and albumin concentrations, and alkaline phosphatase activity, as described.^39^ Plasma calcium was adjusted for variations in albumin concentrations using the formula: ((albumin‐mean albumin) × 0.02) + calcium), as described.^39^

### Statistical analysis

Statistical analysis was performed using Microsoft Excel 2010 (Microsoft Corp., Redmond, WA, USA) and GraphPad Prism (GraphPad Software, Inc., La Jolla, CA, USA). Significance of differences was assessed by unpaired two‐tailed Student's *t* test, or Fisher's exact test^37^; *p *< 0.05 was considered significant.

### Mapping studies, genome sequencing, Sanger DNA sequence analysis and amplification‐refractory mutation system–PCR

Genomic DNA was extracted from ear or tail biopsies as described.^34^ and amplified by PCR for genomewide mapping using a panel of 91 SNP loci arranged in chromosome sets, and the products were analyzed by pyrosequencing.^40^ Whole‐genome sequencing was undertaken using DNA from one affected HVF mouse and the two parental strains (C57BL/6J and C3H/HeH), to generate a library, and 100‐nucleotide (nt) paired‐end sequencing generated using an Illumina HiSeq 2000 sequencer as described.^35^ Sequencing data was analyzed using a previously described pipeline.^35^ Briefly, sequences were aligned to the mouse reference genome NCBIM38/mm10 using the Burrows‐Wheeler Aligner. SNPs and small indels were detected using the Genome Analysis Toolkit (GATK) unified GenotypeCaller^41^ with dbSNP version 137 as the background SNP set and default parameters. Only SNPs with mapping quality >100 and read depth >3 (“high confidence SNPs”) were considered further, and these were functionally annotated using next‐generation sequencing (NGS)‐SNP.^35^ High‐confidence SNPs were filtered against a precompiled list found in 17 inbred strains from the Mouse Genome Project^42^ and from the two parental strains. DNA sequence analysis was undertaken by PCR amplification using gene‐specific primers for individual exons and adjacent splice sites and Taq PCR Mastermix (Qiagen, Crawley, UK), and the DNA sequence of the PCR products determined using BigDye terminator reagents and an ABI 3100 sequencer (Life Technologies, Carlsbad, CA, USA).^34^ Amplification‐refractory mutation system (ARMS)‐PCR was undertaken for further studies of a variant in *Map3k7* in 24 mice, as described.^43^

## Results

### Identification of HVF mice and inheritance as an autosomal dominant trait

**Figure 1:**
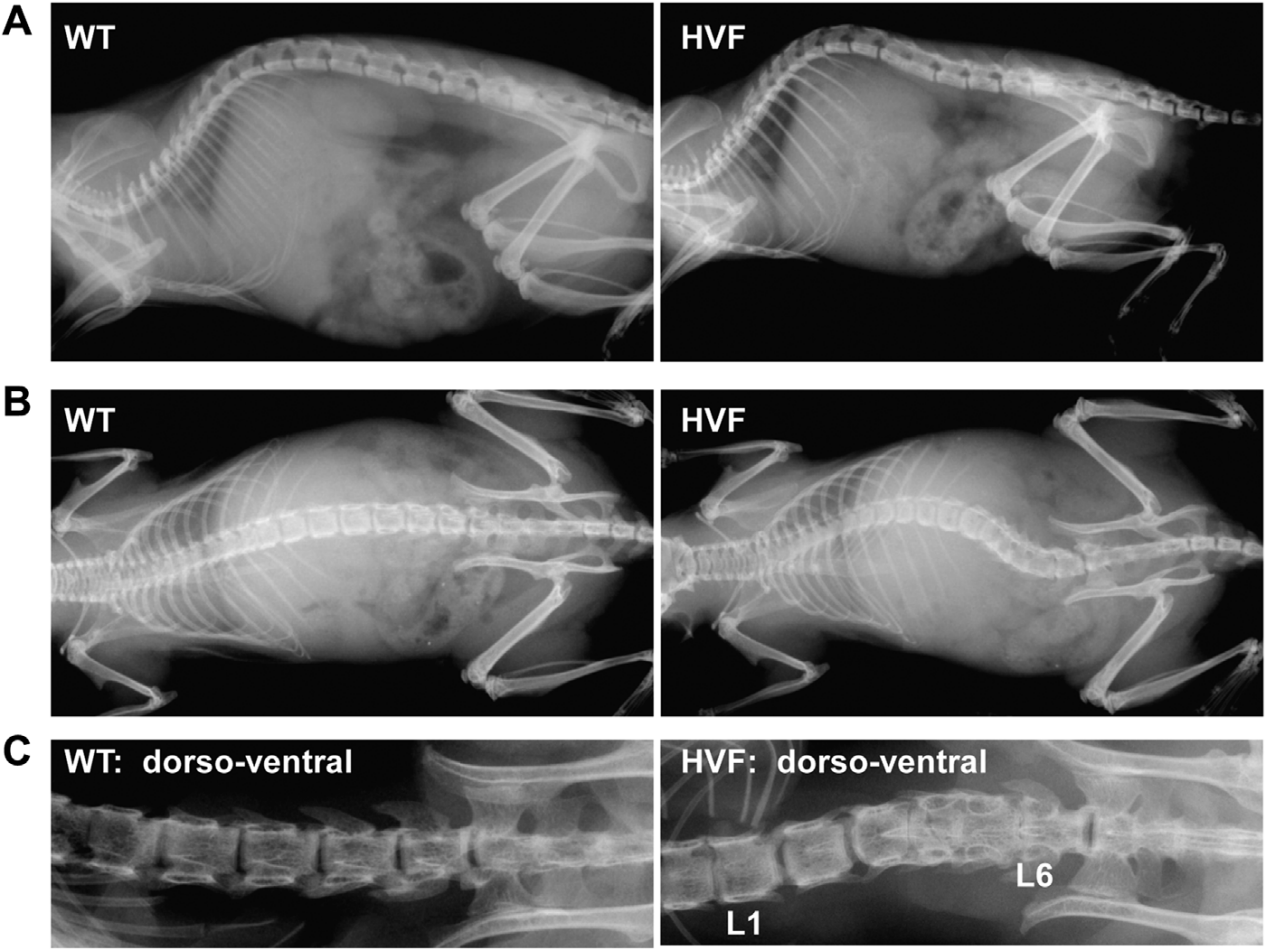
Radiographic analysis of WT littermates and HVF female mice at 12 weeks. Plain radiographs showing (*A*) kyphosis and (*B*) scoliosis in HVF mice. (*C*) Radiographs of dissected vertebrae from WT littermate and HVF mice showing scoliosis and fusion of lumbar vertebrae (L_3_ to L_6_) in HVF mice. WT = wild‐type.

**Figure 2:**
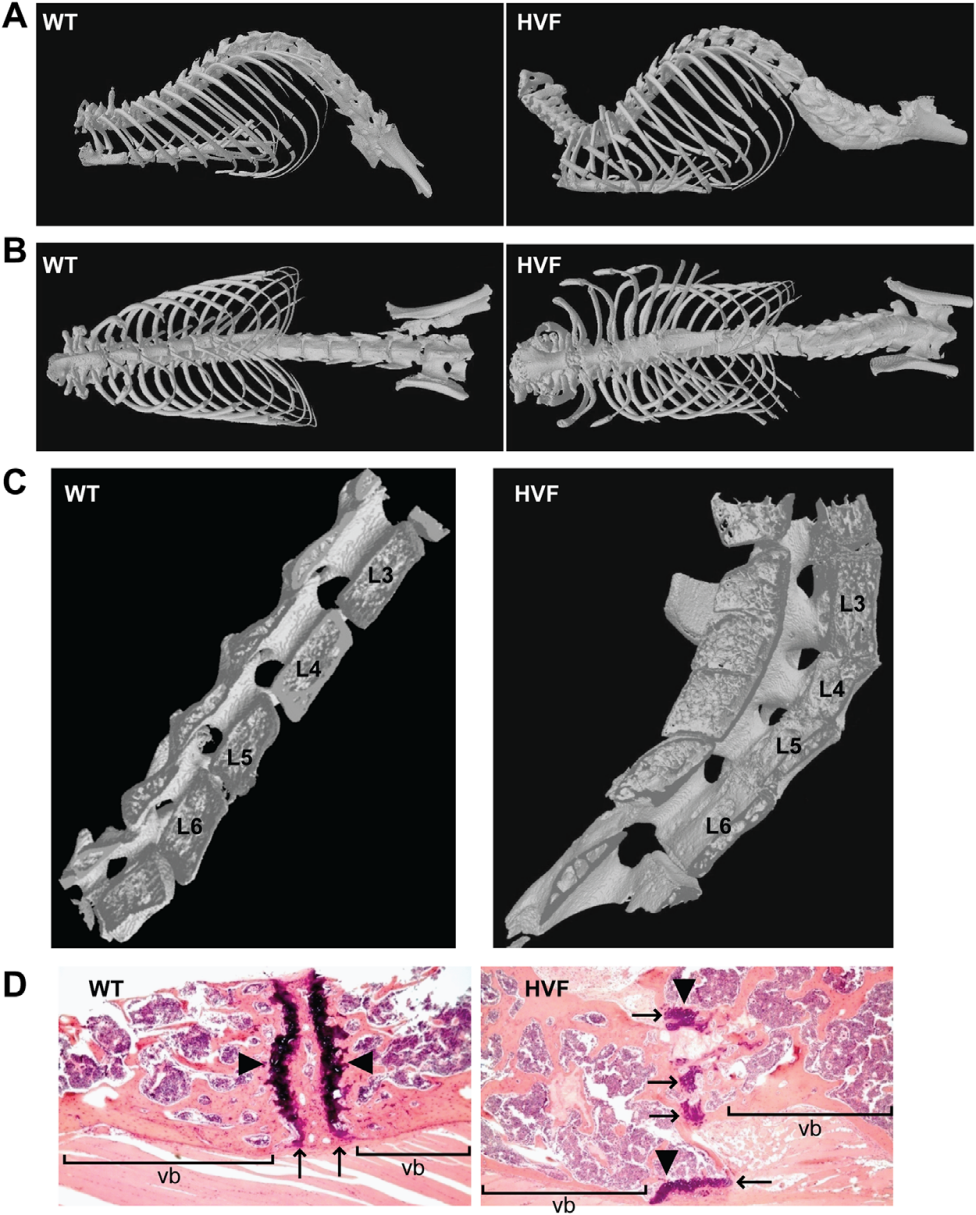
μCT scan and histology analysis of WT littermates and HVF female mice at 12 weeks. μCT scans showing (*A*) kyphosis, (*B*) scoliosis, and (*C*) fused lumbar vertebrae (L_3_ to L_6_) in HVF mice. (*D*) Histology of lumbar vertebrae stained with hematoxylin and eosin, showing irregularities of the ends of the vb with displacement of intervertebral discs (arrows) and ossification centers (arrowheads) in HVF mice. vb = vertebral body; WT = wild‐type.

Radiography analysis of 12‐week‐old progeny derived by mating of an ENU‐mutagenized male C57BL/6J mouse with a wild‐type (WT) C3H/HeH female mouse identified a female mutant with fused lumbar vertebrae (L_2_ and L_3_) and kyphosis (Fig. 1 *A*). Normal mating of this affected female mouse with WT C3H/HeH male mice was repeatedly unsuccessful, because the back deformities, due to the kyphoscoliosis, hindered mounting. In vitro fertilization, utilizing sperm from a WT C3H/HeH male mouse, was therefore used to generate progeny for inheritance testing, and radiography analysis of the 52 (25 male and 27 female) progeny at 12 weeks revealed that 23 (eight males and 15 females) (ie, 44%) were affected with fusion of two to four lumbar vertebrae, consistent with an autosomal dominant inheritance. The phenotype was designated as hereditary vertebral fusion (HVF), and the locus as *Hvf*. HVF was associated with kyphosis in 30% of affected mice, scoliosis in 17%, and kyphoscoliosis in 30% of mice (Fig. 1 *B*, *C*). The affected mice did not have dysmorphology or other radiological abnormalities and were therefore representative of nonsyndromic kyphoscoliosis. In addition, inspection of these mice did not detect any gross morphological abnormalities at earlier ages. μCT scanning analysis confirmed the occurrence of the spinal abnormalities of kyphosis (Fig. 2 *A*) and scoliosis (Fig. 2 *B*) associated with fusion of the lumbar vertebrae (Fig. 2 *C*), which was consistent with a pattern of blocked vertebrae due to failure of segmentation. Histology of the lumbar vertebrae revealed irregularly shaped vertebral bodies and displacement of intervertebral discs and ossification centers in HVF mice (Fig. 2 *D*). The severity of the HVF phenotype was similar in males and females and the proportion of males that were affected (32%) and females that were affected (56%) did not differ significantly (Fisher's exact test, *p* = 0.103), consistent with the autosomal dominant inheritance of HVF. Analysis of plasma calcium, phosphate, and albumin concentrations and alkaline phosphatase activity did not reveal any differences between HVF mice and unaffected littermates (data not shown).

### Phenotypic assessment of HVF mice

**Figure 3:**
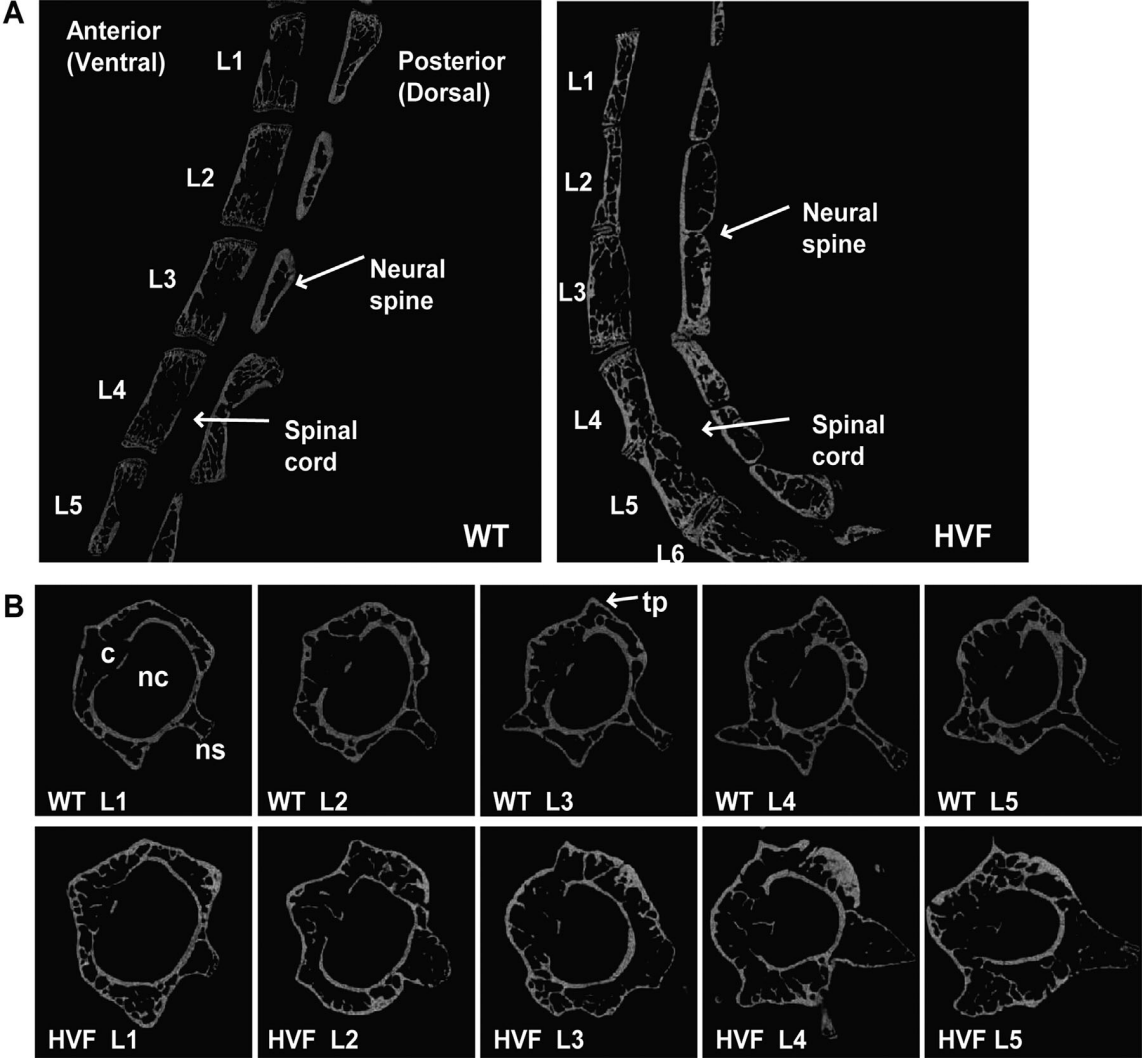
μCT cross‐sectional analysis of spinal column from 12‐week‐old WT littermates and HVF female mice. (*A*) Cross‐section of spinal column showing fusion of L_4_ and L_5_, and abnormalities of the neural spine between L_3_ and L_4_ in HVF mice. (*B*) Cross‐section of lumbar vertebrae from WT littermates and HVF mice, revealing altered shape of nc, wider ns, and bone formation near tp in HVF mice. WT = wild‐type; nc = neural canal; ns = neural spine; tp = transverse process.

Further phenotype analysis using DXA and μCT analysis was undertaken. DXA was performed on 30 mice, which consisted of 20 WT littermates (13 males and seven females) and 10 HVF mice (three males and seven females), aged 12 weeks. Body weight was significantly reduced by >25% in the HVF mice (mean ± SD of WT versus HVF females = 28.66 ± 1.58 g versus 21.03 ± 0.78 g, *p *< 0.001; and WT males = 35.02 ± 2.43 g, with each of the three HVF male mice being −5 SD below the WT mean, and this was associated with a >20% decrease in lean mass (WT versus HVF females = 20.62 ± 1.68 g versus 16.15 ± 0.78 g, *p *< 0.001; and WT males = 27.14 ± 1.87 g with each of the three HVF male mice being −4 to −5 SD below the WT mean) and a >40% decrease in fat mass (WT versus HVF females = 6.27 ± 1.18 g versus 3.66 ± 0.46 g, *p *< 0.001; and WT males = 7.9 ± 2.7 g with each of the three HVF male mice being −1 to −2 SD below the WT mean). Whole‐body bone mineral density (BMD) was similar in female HVF mice compared to WT mice (WT versus HVF = 61.1 ± 2.4 mg/cm^2^ versus 58.4 ± 2.3 g/cm^2^) and ∼8% lower in male HVF compared to WT (WT versus HVF = 61.0 ± 0.9 g/cm^2^ with each of the three HVF male mice being −3 to −6 SD below the WT mean). μCT analysis was undertaken on the lumbar vertebrae from 11 mice, which comprised five WT females and six HVF females (Fig. 3). Cross‐sectional analysis of the vertebral column from WT and HVF mice (Fig. 3 *A*) revealed HVF mice to have generalized disc narrowing, fusion of lumbar vertebrae, and displacement with compression of the neural spine adjacent to regions with dorsal disc narrowing. Cross‐sectional analysis of individual lumbar vertebrae revealed the HVF mutant to have distorted transverse processes, and wider neural spine and bone formation in the spaces between L_3_, L_4_, and L_5_ (Fig. 3 *B*). In addition, L_3_ to L_5_ from the HVF mutant had distorted neural canals, and the lower ends of L_2_ to L_5_ had an abnormal orientation due to scoliosis in this region (Fig. 3 *B*). Additional quantitative cross‐sectional analysis of the first and second lumbar vertebrae (Fig. 3 *B*) from WT (*n* = 13 males and *n* = 13 females) and HVF mice (*n* = 5 males and *n* = 9 females) revealed no significant differences in trabecular bone volume, trabecular thickness, or bone density. X‐ray, DXA, and μCT analyses did not reveal any other bone phenotypes apart from the vertebrae.

### Mapping of the *Hvf* locus to chromosome 4A3 and genome sequencing

**Figure 4:**
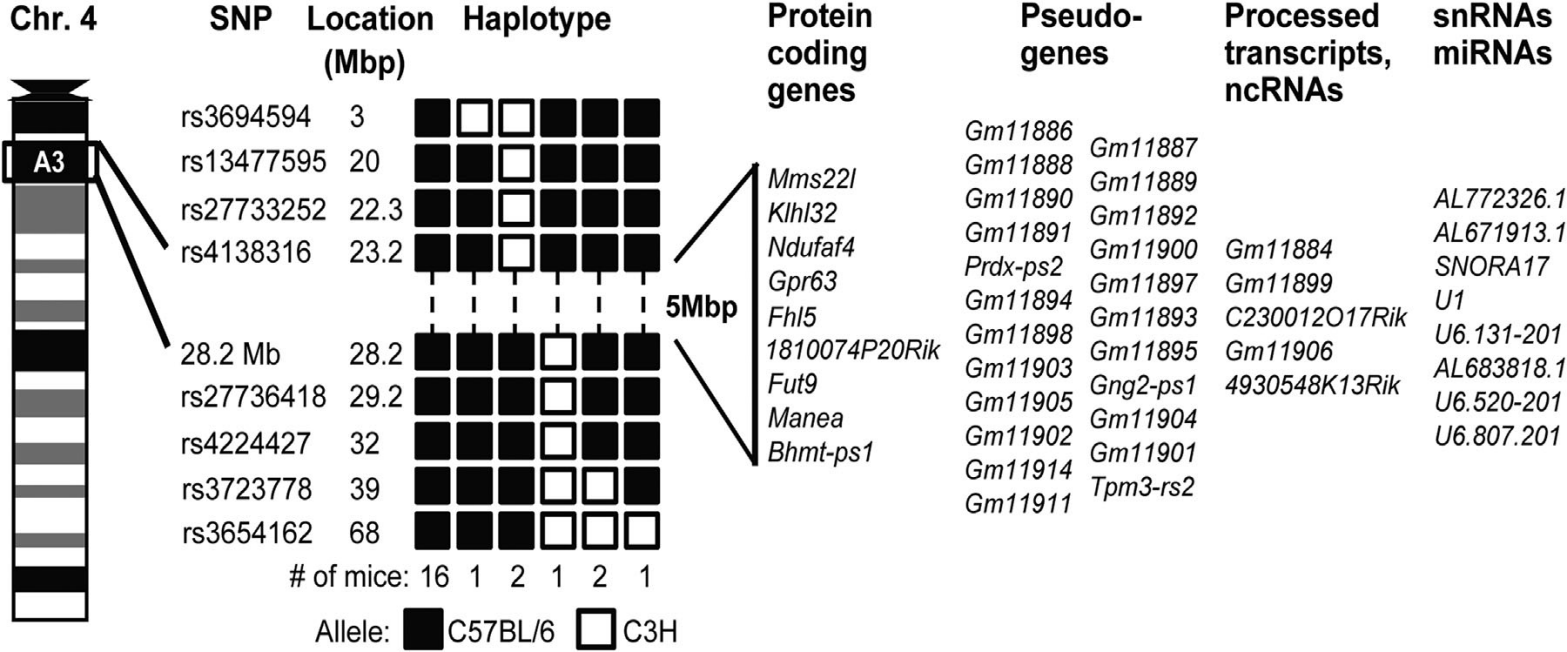
Haplotype analysis using chromosome 4 SNPs in mice affected with HVF. The *Hvf* locus is inherited with C57BL/6 alleles and is located in a 5‐Mb interval between the centromeric SNP rs4138316 and the telomeric microsatellite locus at the 28.2‐Mb position. The region contains nine protein coding genes that were studied for mutations, 24 pseudogenes, two processed transcripts, three large intergenic ncRNAs, five snRNAs, and three miRNAs. ncRNA = noncoding RNA; snRNA = small nuclear RNA; miRNA = microRNA.

Genomewide mapping studies using 97 SNPs and DNA samples from 23 HVF (15 females and eight males) affected mice mapped the *Hvf* locus to chromosome 4. HVF was found to cosegregate with C57BL/6 alleles identified by nine SNPs from chromosome 4 (Fig. 4) (LOD score > +6.5), and no other locus was found to segregate with the HVF phenotype. An examination of the haplotypes helped to further localize the *Hvf* locus; 16 HVF mice had inherited non‐recombinant chromosome 4 haplotypes, whereas seven HVF mice had inherited recombinant haplotypes. The recombinant haplotypes in three HVF mice helped to define the critical 5‐Mb interval (Fig. 4); because two HVF mice had recombinants between the disease locus (*Hvf*) and the centromeric SNPs, including rs4138316, and another mouse had a recombination between *Hvf* and the telomeric loci, including the microsatellite locus at map position 28.2 Mb. These results locate *Hvf* to a 5‐Mb interval flanked centromerically by rs4138316 and telomerically by the microsatellite locus at the 28.2‐Mb position. This interval could not be refined further because strain‐specific polymorphic loci within this region are not available. This interval contains 46 genes or likely expressed transcripts which include: the nine known protein coding genes (kelch‐like 32 [*Klhl32*], NADH dehydrogenase‐ubiquinone‐1‐alpha subcomplex assembly factor 4 [*Ndufaf4*], G protein‐coupled receptor 63 [*Gpr63*], four and a half LIM domains 5 [*Fhl5*], fucosyltransferase 9 [*Fut9*], mannosidase endo‐alpha [*Manea*], betaine‐homocysteine methyltransferase pseudogene‐1‐pseudogene [*Bhmt‐ps1*], MMS22‐like DNA repair protein [*Mms22L*], and RIKEN cDNA 1810074P20 gene [*1810074P20Rik*]) (Fig. 4); two processed transcripts; three microRNAs; five small nuclear RNAs; three large intergenic noncoding RNAs; and 24 pseudogenes (Supporting Table  1). An analysis of these genes and likely expressed transcripts did not reveal any links with the molecular pathways that are known to cause kyphosis or patterning defects, such as the Notch pathway, and we therefore carried out whole‐genome sequencing. Approximately 95% of the 5‐Mb candidate interval was covered by sequencing data, with an average depth of 11×. This did not identify any nucleotide variants within the coding regions and splice junctions any of the nine coding genes, the two processed transcripts, three microRNAs, five small nuclear RNAs, three large intergenic noncoding RNAs, or 24 pseudogenes within the 5‐Mb candidate interval, or up to 2 Mb telomeric or centromeric to the candidate interval. This was confirmed by Sanger DNA sequence analysis of the coding regions, splice junctions and 5′ untranslated regions of the nine known coding genes, and of the five small nuclear RNAs, which did not reveal any abnormalities. Six intergenic and three intronic SNPs were present within the candidate region; however, none of these SNPs were found to be within conserved sequences between human, rat, and chimp genomes (Vista Genome Browser; http://pipeline.lbl.gov/cgi-bin/gateway2), or within any regulatory regions in ENCODE (https://www.encodeproject.org/). Genome sequencing identified a novel, high‐confidence coding variant in the mitogen‐activated protein kinase kinase kinase 7 (*Map3k7*) gene, which was located ∼3.8 Mb telomerically of the critical interval. This variant, which was a C>A transversion at c.702 of *Map3k7* that predicted the missense mutation Ala179Asp, was studied, using ARMS‐PCR, for cosegregation with the HVF phenotype in 24 mice (nine HVF and 15 unaffected littermates). This *Map3k7* variant did not cosegregate with the HVF phenotype, because five of 15 unaffected mice were found to harbor the variant and one of nine affected HVF mice did not harbor the variant (data not shown), consistent with this *Map3k7* variant being located outside the critical interval. Thus, the causative mutation for HVF is likely to involve the regulatory region of one the 46 genes within the critical interval, or possibly a regulatory region that acts over a longer range to alter the expression of a gene outside the critical interval.

## Discussion

Our study has established an ENU‐induced mutant mouse model, HVF, for an autosomal dominant form of nonsyndromic kyphoscoliosis that is associated with fusion of lumbar vertebrae, and reductions in lean mass and fat mass, and this has some similarities to a form of human congenital scoliosis referred to as block vertebrae.^9^ Congenital scoliosis in man can be classified into two main patterns of presentation, namely: (i) failure of formation due to the presence of hemivertebrae and wedged vertebrae; and (ii) failure of segmentation which consists of unilateral unsegmented bars with and without hemivertebrae, and bilateral failure of segmentation (block vertebrae).^9^ The HVF mouse model resembles the failure of segmentation (block vertebrae) pattern (Figs. 1 and 2; Table 1) which has been reported in ∼3% of 251 patients with congenital scoliosis,^44^ and to have a prevalence of approximately 0.5 to 1 per 1000 individuals.^9^ Thus, identifying the genetic defect causing HVF may help to identify a component in the complex pathways regulating vertebral formation, which is likely to involve the expression of multiple genes. Indeed, studies of other mouse mutants presenting with abnormalities in the axial skeleton including tail kinks, kyphosis, and scoliosis have revealed multiple conserved chromosomal loci harboring genes that are likely to be candidates for human kyphosis and scoliosis^8^, ^45^; however, it is clear from their polygenic nature that no single mouse mutant identified thus far can serve as a complete model for investigating these disorders.

**Table 1:** Phenotypic Features of HVF Mice, CSmo, Heterozygous Knockout Mouse Models (*Mesp2* ^+/−^, *Hes7* ^+/−^, and *Dll1* ^+/−^), ISR, and CShu

	Rodent models						Human
	HVF	CSmo^a^	*Mesp2* ^+/–^ ^b^	*Hes7* ^+/–^ ^b^	*Dll1* ^+/–^ ^b^	ISR^c^	CShu^d^
Derivation of mutation	ENU	CO	TKO	TKO	TKO	SN	SN
Inheritance	Ad	Ni	Ad (rp)	Ad (rp)	Ad (rp)	Ar	?
Kyphosis	+	–	– (–)	– (–)	– (–)	+	†
Scoliosis	+	+	– (–)	– (+)	– (–)	–	+
Fusion of vertebrae	+	–	– (+)	– (+)	– (–)	+	+
Segmentation defects	+	–	– (+)	+ (+)	+ (+)	+	+
Wedged vertebrae	+	+	– (–)	– (–)	– (–)	+	+
Hemivertebrae and bars	–	+	– (–)	– (–)	– (–)	–	+
Narrow intervertebral spaces	+	–	? (?)	? (?)	? (?)	+	?
Body weight	↓	?	? (?)	? (?)	? (?)	?	?
Lean mass	↓	?	? (?)	? (?)	? (?)	?	?
Fat mass	↓	?	? (?)	? (?)	? (?)	?	?
BMD	N/↓	N	? (?)	? (?)	? (?)	?	?

CSmo = congenital scoliosis mouse model; ISR = Ishibashi Rat; CShu = human congenital scoliosis‐block vertebrae; ENU = N‐ethyl‐N‐nitrosourea induced model; CO = carbon monoxide induced; TKO = targeted knock‐out; SN = spontaneous naturally occurring; Ad = autosomal dominant; (rp) = reduced penetrance; Ni = not inherited; Ar = autosomal recessive;  ? = not reported; + = present; – = absent; † = patients with kyphosis excluded from study; N = normal; ↓ = reduced; ↑ = increased.

^a^ Previously reported (Ishibashi(47)).

^b^ Previously reported (Sparrow and colleagues(51)), penetrance was increased with exposure to hypoxia. Symbols without parentheses denote phenotype with normoxia; symbols in parentheses denote phenotype with hypoxia.

^c^ Previously reported (Ishibashi(46) and Moritake and colleagues(48)).

^d^ Previously reported (McMaster and Ohtsuka(44)).

The phenotype of HVF mice also has some similarities to two other rodent models; these are the Ishibashi rat (ISR) model which arose spontaneously during inbreeding of agouti rats originating from breeding of a Wistar female rat to a wild rat,^46^ and the congenital scoliosis mouse model (CSmo) developed by exposing pregnant DBA/1J mice to 600 ppm of carbon monoxide on day 9 of gestation^47^ (Table 1). Thus, the ISR model has similarities to HVF mice in having kyphosis, that is associated with segmentation defects affecting mainly the lumbar area, as well as narrowing of intervertebral spaces, irregularity of adjacent ends of vertebral bodies, and wedging and complete bony fusion of adjacent vertebral bodies.^48^, ^49^ However, the ISR model is suggested to have an autosomal recessive inheritance^50^ and although reduced expression of *Hox10* and *Hox11* have been reported,^49^ a mutation has not yet been identified. Details of body weight, lean mass, fat mass, and BMD content were not reported in the ISR model.^48^ The CSmo model has few similarities to the HVF mice other than scoliosis which may involve the lumbar vertebrae^47^; however, the CSmo model has scoliosis in association with hemivertebrae and bars that may affect cervical, thoracic, or lumbar vertebrae.^47^ Details of body weight, lean and fat mass, and BMD were not reported in the CSmo model.^47^ Three other knockout mouse models with an allelic deletion of genes encoding members of the Notch signaling pathway have been reported to develop vertebral defects (Table 1). These mice have a heterozygous loss of the mesoderm posterior protein 2 (*Mesp2* ^+/−^), hairy and enhancer of split 7 (*Hes7* ^+/−^), and delta‐like 1 (*Dll1* ^+/−^) genes, and all three mouse models have been reported to develop autosomal dominantly inherited vertebral defects with low penetrance.^51^ However, the penetrance and severity of these defects, in embryos with an allelic deletion (ie, *Mesp2* ^+/−^, *Hes7* ^+/−^, or *Dll1* ^+/−^), were increased by exposure to hypoxic conditions, and the resulting defects included missing pedicles and rib abnormalities.^51^ These findings indicate that the *Mesp2* ^+/−^, *Hes7* ^+/−^, and *Dll1* ^+/−^ mice do not have any similarities to the HVF mouse model, which develops autosomal dominant kyphoscoliosis with high penetrance without any requirement for environmental challenge such as hypoxia. Null mutations of *Tbx6*, a key regulator of the Notch signaling pathway, cause congenital scoliosis in humans and rats.^27^, ^52^, ^53^, ^54^ However, the vertebral defects may occur in the cervical, thoracic, or lumber spine, in contrast to *Hvf* mice, in which the defects occurred only in L_2_ to L_5_ vertebrae. Thus, our results provide a new rodent model for hereditary lumbar scoliosis. TBX6 is expressed throughout the developing axial skeleton, with differential spatial expression patterns at different time‐points, as vertebral segmentation proceeds in a cranial to caudal direction.^55^ We hypothesize that a noncoding mutation is the cause of the *Hvf* phenotype, likely through mutation of a promoter region. The cellular effect of the mutation is therefore likely to be one of altered regulation of a pathway, either in a time‐specific or space‐specific manner, rather than disruption of a whole pathway, as is the case with TBX6 null mutations. Thus, if cranial‐caudal development of the spine is either terminated at a different time or delayed, then this may be more likely to induce defects in the caudal part of the spine rather than in the cranial regions or throughout, or if spatial specificity is disrupted, then a small specific set of vertebrae may be affected. The time‐sensitive nature of vertebral segmentation is demonstrated by *Mbtps1* conditional knockout mice, in which *Mbtps1* was knocked‐out at embryonic day 8.5 (E8.5) in the caudal region of the embryo. These mice developed fusion of the lower lumbar vertebrae, in a similar pattern to *Hvf* mice.^56^

Our studies mapped the *Hvf* locus to a 5‐Mb interval on chromosome 4A3 (Fig. 4) and the syntenic region (23.2 to 28.2 Mb) in humans (94.2 to 98.8 Mb) is on chromosome 6q16.1. Genetic mapping studies in humans have revealed loci for scoliosis and/or kyphosis on chromosomes 8, 17p11.2, and 19q13.3, and these do not correspond to the syntenic region to mouse chromosome 4A3, thereby indicating that further studies of HVF mice may also identify a gene for kyphoscoliosis in humans. Indeed, kyphosis and/or scoliosis are likely to have the involvement of many different genes as well as representing common clinical endpoints for a number of diseases that have different pathogenetic mechanisms,^8^, ^45^ such as the CHARGE syndrome, which is associated with later‐onset scoliosis in more than 60% of patients.^11^ The CHARGE syndrome is due to mutations of the chromodomain‐helicase‐DNA‐binding protein 7 (*CHD7*) gene^11^ and a *CHD7* polymorphism (rs4738824, chromosome 18q12) has been associated with susceptibility to idiopathic scoliosis in a group of families of European descent.^15^ However, *CHD7* gene abnormalities have not been identified in other studies of scoliosis pedigrees, thereby indicating the likely involvement of other genes in the etiology of the idiopathic forms of kyphosis and/or scoliosis.

Analysis of the genes within the *Hvf* candidate interval (Supporting Table  1) did not reveal any links with molecular pathways known to be involved with kyphosis, patterning defects, or vertebral fusion, such as the Notch pathway. However, the candidate interval contains *Ndufaf4*, which encodes a protein that is required for assembly of complex I of the mitochondrial respiratory chain, and affected individuals from a family with isolated mitochondrial complex I deficiency due to homozygous mutations in *NDUFAF4* have been reported to have kyphosis; however, this occurred in conjunction with severe metabolic acidosis, encephalopathy, and death in infancy, whereas heterozygous parents and siblings were unaffected.^57^ HVF mice did not have premature death or encephalopathy; moreover, the HVF phenotype was inherited in an autosomal dominant manner, thereby indicating that HVF is unlikely due to defects of *Ndufaf4*. Furthermore, mice with a heterozygous deletion of *Ndufs4*, that encodes one of the proteins within complex I, and therefore has isolated complex I deficiency, were indistinguishable from WT mice, with no kyphoscoliosis reported.^58^ The candidate interval also contains three microRNAs; however, these are all novel microRNAs whose targets are unknown.

We also investigated *Map3k7*, a coding variant (Ala179Asp) that was located outside the critical interval, because Map3k7 (also known as TGF‐β‐activated kinase 1 [TAK1]) is a member of the signaling pathway that links TGF‐β and bone morphogenetic protein (BMP) with activation of the p38 MAPK pathway, which plays a critical role in bone growth. However this *Map3k7* variant did not cosegregate with the HVF phenotype, and involvement of this missense Map3k7 variant in causing HVF is unlikely, and consistent with other observations from mutant mouse and human disease studies. Thus, homozygous *Map3k7* knockout mice are embryonically lethal, and heterozygous *Map3k7* knockout mice have no phenotype.^59^, ^60^ Moreover, osteoblast‐specific *Map3k7* knockout mice developed clavicular hypoplasia and delayed closure of the fontanelles, similar to the human disorder of cleidocranial dysplasia, reduced trabecular bone, and a moderate decrease in body weight, but did not develop vertebral defects.^61^ Conversely, osteoclast precursor‐specific *Map3k7* knockout mice displayed skull overgrowth and increased trabecular bone, but again did not develop any vertebral abnormalities.^62^, ^63^ Furthermore, heterozygous mutations in *MAP3K7* in humans cause the syndromic skeletal disorders of cardiospondylocarpofacial syndrome and frontometaphyseal dysplasia.^64^, ^65^ These studies illustrate that Map3k7 plays an important role in bone development, and it is possible that the HVF mice harbor a noncoding mutation within the critical interval that alters the regulation of Map3k7 specifically in developing vertebrae. Indeed, a recent study has suggested that familial idiopathic kyphoscoliosis/scoliosis in a series of seven families, in whom a critical interval of 3.5 Mb on chromosome 5p was previously defined, may be due to a noncoding mutation within a regulatory region that affected the expression of an unknown target gene(s).^66^ Because there are no known links between any of the genes within the *Hvf* candidate interval and pathways involved with kyphosis, patterning defects, or vertebral fusion, the genetic defect causing the HVF phenotype may reveal novel biological mechanisms involved with these processes.

In humans, a number of inherited diseases, with autosomal dominant and recessive, and X‐linked inheritances, have been described to be due to noncoding mutations. These include triphalangeal thumb/preaxial polydactyly (autosomal dominant with variable penetrance), due to mutations in the ZPA regulatory sequence, a long‐range cis‐acting regulator of *Sonic Hedgehog* (*SHH*) gene expression^67^, ^68^, ^69^; and autosomal recessive isolated pancreatic agenesis due to noncoding mutations downstream of *pancreas‐specific transcription factor 1a* (*PTF1A*).^70^ Such mutations are frequently large deletions or duplications, but may also consist of single‐nucleotide mutations, similar to those most frequently induced by ENU, as found in isolated pancreatic agenesis, and recently described in the promoter region of *ovo‐like zinc finger 2* (*OVOL2*) in families with autosomal‐dominant corneal endothelial dystrophies.^71^ Mutations upstream of *PTF1A* were found to reduce the expression of *PTF1A*, whereas mutations in the *OVOL2* promoter were able to induce *OVOL2* expression, likely leading to aberrant ectopic *OVOL2* expression in the developing cornea.^70^, ^71^ Either of these two molecular mechanisms could account for the dominant disease presentation in *Hvf* mice, eg, through a dosage effect due to a reduction in the level of a critical transcript, or through ectopic induction of a transcript, and either of these may be time and/or spatially specific. Future work to identify the *Hvf* causative mutation will focus on analyses of the six intergenic and three intronic noncoding variants in transcriptional assays, such as luciferase assays, similarly to those undertaken for PTF1A and OVOL2‐associated noncoding variants.^70^, ^71^ To determine the specific role of the variants in somitogenesis, these assays may need to be undertaken in a somite cell‐line, such as cells derived from pluripotent stem cells,^72^ or in bone‐specific cells such as chondrocytes.

In summary, our study has established an ENU‐induced mouse model for autosomal‐dominant congenital scoliosis and identification of the causative genetic defect will help in further elucidating the molecular mechanisms associated with congenital scoliosis due to segmentation defects.

## Disclosures

All authors state that they have no conflicts of interest.

## Supporting information

Supporting Table S1.
