# Supplementary material for: An N‐Ethyl‐N‐Nitrosourea (ENU) Mutagenized Mouse Model for Autosomal Dominant Nonsyndromic Kyphoscoliosis Due to Vertebral Fusion
Source: JBMR Plus. 2018 Mar 8;2(3):154–63. doi: 10.1002/jbm4.10033 (PMC6124210; doi:10.1002/jbm4.10033)
Supplement: Supplementary file 1 — Supporting Table S1. [file JBM4-2-154-s001.docx]

Supplementary Table 1. Genes and likely expressed transcripts in the *Hvf* candidate interval

| Gene | Position | Name | Function |
| --- | --- | --- | --- |
| Gm11884 (partial) | 23,260,784-23,392,503 | Known long interspersed non-coding RNA | No protein product |
| Gm11886 | 23,422,513-23,422,815 | Novel pseudogene | No protein product |
| Gm11887 | 23,440,018-23,440,247 | Novel pseudogene | No protein product |
| Gm11888 | 23,484,820-23,486,020 | Ribosomal protein S2 (Rps2) pseudogene | No protein product |
| Gm11889 | 23,517,362-23,518,440 | LUC7-like 2 (S. cerevisiae) (Luc7l2) pseudogene | No protein product |
| AL772326.1 | 23,626,656-23,626,745 | Novel miRNA |  |
| Gm11890 | 23,659,928-23,660,344 | Ribosomal protein L27a (Rpl27a) pseudogene | No protein product |
| Gm11892 | 24,238,100-24,239,301 | Tubulin beta 2 (Tubb2) pseudogene | No protein product |
| Gm11891 | 24,423,477-24,425,192 | Ribosomal protein L13 (Rpl13) pseudogene | No protein product |
| Mms22l | 24,496,451-24,602,950 | Methyl methanesulfonate-sensitivity protein 22-like | DNA repair protein |
| Klhl32 | 24,612,554-24,851,124 | Kelch-like 32 (Drosophila) | Unknown function |
| AL671913.1 | 24,689,646-24,689,726 | Novel miRNA within Klhl32 | No known targets |
| Gm11899 | 24,792,680-24,800,310 | Novel antisense processed transcript within Klhl32 | No protein product |
| Ndufaf4 | 24,898,083-24,905,001 | NADH dehydrogenase-ubiquinone complex 1, assembly factor 4 | Assembly factor for complex 1 of mitochondrial respiratory chain |
| C230012O17Rik | 24,960,790-24,967,449 | Known long interspersed non-coding RNA | No protein product |
| Gpr63 | 24,966,407-25,009,223 | G protein-coupled receptor 63 | Orphan receptor |
| Gm11906 | 24,966,517-25,271,099 | Novel processed transcript | No protein product |
| Gm11900 | 25,086,849-25,087,200 | Novel pseudogene | No protein product |
| Fhl5 | 25,199,908-25,242,876 | Four and a half LIM domains 5 | Stimulator of transcriptional activity of CREM |
| 1810074P20Rik | 25,248,600-25,281,821 | UFM1-specific ligase 1 | Protein ligase, mediates ufmylation of target proteins |
| Prdx6-ps2 | 25,307,830-25,308,523 | Peroxiredoxin 6 pseudogene 2 | No protein product |
| Gm11897 | 25,353,702-25,354,529 | ZW10 homolog (Drosophila), centromere/kinetochore protein (Zw10) pseudogene | No protein product |
| Gm11894 | 25,419,349-25,419,676 | GLE1 RNA export mediator-like (yeast) (Gle1l) pseudogene | No protein product |
| SNORA17 | 25,513,451-25,513,575 | Small nucleolar RNA | Predicted to guide pseudouridylation of 28S rRNA |
| Gm11896 | 25,538,850-25,539,669 | KRR1, small subunit (SSU) processome component, homolog (yeast) pseudogene | No protein product |
| Gm11893 | 25,541,791-25,542,281 | Telomerase binding protein, p23 pseudogene | No protein product |
| U1 | 25,566,660-25,566,822 | U1 spliceosomal RNA | Small nuclear RNA component of U1 small nuclear ribonucleoprotein |
| Fut9 | 25,609,332-25,800,244 | Fucosyltransferase 9 | Last step in synthesis of Lewis X antigen in Golgi apparatus |
| U6.131-201 | 25,819,671-25,819,773 | U6 spliceosomal RNA | Small nuclear RNA component of U6 small nuclear ribonucleoprotein |
| Gm11898 | 25,854,515-25,854,899 | G protein-coupled receptor 155 (Gpr155) pseudogene | No protein product |
| AL683818.1 | 25,861,239-25,861,348 | Novel miRNA | No known targets |
| Gm11895 | 25,867,989-25,868,455 | Hematological and neurological expressed sequence 1 (Hn1) pseudogene | No protein product |
| U6.520-201 | 25,940,365-25,940,470 | U6 spliceosomal RNA | Small nuclear RNA component of U6 small nuclear ribonucleoprotein |
| Manea | 26,324,506-26,346,891 | Mannosidase, endo-alpha | Processing of oligosaccharides in Golgi apparatus |
| Bhmt-ps1 | 26,368,984-26,370,207 | Betaine-homocysteine methyltransferase, pseudogene 1 | Evidence of transcript expression but not of protein expression |
| Gm11903 | 26,442,461-26,443,187 | Ribosomal protein S6 (Rps6) pseudogene | No protein product |
| Gng2-ps1 | 26,501,443-26,501,659 | G protein gamma 2 subunit, pseudogene 1 | No protein product |
| Gm11905 | 26,593,490-26,594,167 | Peroxiredoxin 3 (Prdx3) pseudogene | No protein product |
| 4930548K13Rik | 26,635,820-26,705,449 | Novel long interspersed non-coding RNA | No protein product |
| Gm11904 | 27,126,262-27,126,509 | Novel pseudogene | No protein product |
| Gm11902 | 27,222,135-27,222,404 | DEP domain containing 6 (Depdc6) pseudogene | No protein product |
| Gm11901 | 27,543,178-27,543,614 | Ribosomal protein S2 (Rps2) pseudogene | No protein product |
| U6.807-201 | 27,700,671-27,700,777 | U6 spliceosomal RNA | Small nuclear RNA component of U6 small nuclear ribonucleoprotein |
| Gm11914 | 27,867,149-27,867,665 | Nucleosome binding protein 1 (Nsbp1) pseudogene | No protein product |
| Tpm3-rs2 | 27,996,583-27,996,905 | Tropomyosin 3, related sequence 2 (pseudogene) | No protein product |
| Gm11911 | 28,067,177-28,068,199 | DEAD box polypeptide 18 (Ddx18) pseudogene | No protein product |

LIM: Lin11, Isl-1, Mec-3; CREM: cAMP-responsive element modulator; UFM1: ubiquitin-fold modifier 1; DEAD: Asp-Glu-Ala-Asp
